# Comparative analysis of buds transcriptome and identification of two florigen gene *AkFTs* in *Amorphophallus konjac*

**DOI:** 10.1038/s41598-022-10817-5

**Published:** 2022-04-26

**Authors:** Han Gao, Yan Zhao, Lihua Huang, Yu Huang, Jinjun Chen, Haiyan Zhou, Xuewen Zhang

**Affiliations:** College of Biosciences and Biotechnology, Hunan Agricultural University, Changsha, 410128 China

**Keywords:** Transcriptomics, Plant molecular biology

## Abstract

Leaves and flowers of *Amorphophallus konjac* do not develop simultaneously thus unique features can be elucidated through study of flowering transformation in *A. konjac.* In this study, transcriptome libraries of *A. konjac* leaf buds (LB) and flower buds (FB) were constructed followed by high-throughput sequencing. A total of 68,906 unigenes with an average length of 920 bp were obtained after library assembly. Out of these genes, 24,622 unigenes had annotation information. A total of 6859 differentially expressed genes (DEGs) were identified through differential expression analysis using LB as control. Notably, 2415 DEGs were upregulated whereas 4444 DEGs were downregulated in the two transcriptomes. Go and KEGG analysis showed that the DEGs belonged to 44 functional categories and were implicated in 98 metabolic pathways and 38 DEGs involved in plant hormone signal transduction. Several genes were mined that may be involved in *A. konjac* flower bud differentiation and flower organ development. Eight DEGs were selected for verification of RNA-seq results using qRT-PCR analysis. Two *FLOWERING LOCUS T* (*FT*) genes named *AkFT1* and *AkFT2* were identified though homologous analysis may be the florigen gene implicated in modulation of *A. konjac* flowering. These genes were significantly upregulated in flower buds compared with the expression levels on leaf buds. Overexpression of *AkFT* genes though heterologous expression in *Arabidopsis* showed that the transgenics flowered at a very early stage relative to wild type plants. These findings indicate that *AkFT1* and *AkFT2* function as regulation genes in *A. konjac* flowering development and the two genes may present similar functions during flowering transition.

## Introduction

*Amorphophallus konjac* is a perennial plant and a member of Araceae family. Approximately 163 species of *Amorphophallus* plants have been identified and are widely distributed in China, Japan and Southeast Asia^1^. *Amorphophallus* corm is characterized by large amounts of glucomannan which is often regarded as salutary dietary polysaccharide used in food, medical, health care and other industries^2^. *Amorphophallus* plants are mainly planted in the mountains and hills as a food source and do not compete with cereal crops for land^3^. *Amorphophallus* plants are propagated through asexual methods using mini corm or corm cuttings^4,5^. Asexual propagation is associated with low propagation coefficient, high susceptibility to soft rot and other diseases^6^. Seed propagation is generally considered a better way for crop reproduction. However, *A. konjac* like most *Amorphophallus* species takes more than three years to bloom and they are self-incompatible. Therefore, the seed setting rate in the field is low^7–9^. Furthermore, *A. konjac* exhibits a unique flower development process. Germination of the corm either results in a vegetative leaf or a reproductive flower thus the leaf and flower do not occur concurrently during its growing years. Study of flowering related genes and exploring the molecular mechanism of flowering is important for developing approaches to shorten the flowering time by manipulating flowering-related genes of *A. konjac* thus improving the reproductive efficiency.

Molecular biology research of *A. konjac* is currently limited^10–12^. Lack of reference genome sequence significantly hinders gene mining and molecular breeding of *A. konjac*. Transcriptome analysis is an easier approach to obtain genetic information or expression profiles of genes. Currently, the flowering mechanism of several flowering plants has been preliminarily analyzed through transcriptome analysis, and high amounts of gene information have been obtained. Comparative transcriptome analysis was used to explore various transcription factor families and metabolic pathways involved in flower development in *Cicer arietinum*^13^, *Hypericum perforatum* L.^14^. *Jatropha curcas*^15^ and Wucai (*Brassica campestris* L.)^16^. However, the molecular mechanism of flower formation and flowering related genes in *A. konjac* has not been elucidated.

*FLOWERING LOCUS T* (*FT*) is one of the key genes in the regulation of flowering in plants^17^. *FT* is expressed mainly in leaves and is transported via the phloem to the apical meristem tissue of the stem tip to function in *Arabidopsis thaliana*^18^. *FT* can integrate signals from various flowering regulatory pathways, including endogenous factors and environmental conditions, to regulate the timing of flowering in plants^19^. Previous studies have shown that plant *FT* genes in *A. thaliana*, rice, wheat, and maize have a role in regulating flowering time^20–22^. In addition, *FT* genes can also affect *A. thaliana* seed development, control bulb formation in onion and regulate potato storage organ formation^23–25^.

In this study, Illumina Hiseq high-throughput sequencing technology was used to sequence the transcriptome of *A. konjac* leaf buds and flower buds. The findings from differential expression analysis showed that several genes are implicated in flowering. Two flowering related genes highly homologous to *FT* gene were identified in *A. konjac* and were named *AkFT1* and *AkFT2*, respectively. Overexpression of the two genes in *A. thaliana* through heterologous transformation significantly accelerated flowering in the transgenic plants relative to the wild type plants. These results provide a basis for further study on the molecular mechanism of *A. konjac* flower development process.

## Materials and methods

### Materials

*Amorphophallus konjac* K.Koch plants were grown in Sangzhi (Zhangjiajie, China) under natural conditions. RNA was extracted from leaf primordium and flower primordium for transcriptome analysis.

*Arabidopsis thaliana* ecotype Columbia (sustained in our laboratory) was used as the model plant for gene function analysis. Candidate genes were transformed into *Arabidopsis* through inflorescence infiltration method. *Arabidopsis* seeds were surface-sterilized, germinated on a plate containing Murashige and Skoog (MS) medium with 2% (w/v) sucrose and 0.75% (w/v) agar supplemented with 30 mg/L basta to select transgenic plants. Plants were transferred into pots containing a mixture of topsoil and vermiculite (3:1). The plants were then grown in a growth chamber at 25 °C under a 16-h light/8-h dark photoperiod.

### Construction, sequencing and analysis of cDNA library

Total RNA was extracted from buds using HiPure HP Plant RNA Kit (Magen, China). cDNA libraries were prepared using NEBNext^®^Ultra™ RNA Library Prep Kit for Illumina^®^ (NEB, USA). 1 μg RNA per sample was used as input material for preparation of libraries. The libraries were sequenced using an Illumina Hiseq 2000 platform at Biomarker Technologies Co, LTD (Beijing, China) and paired-end reads of 2 × 100 bp were generated.

Raw data in fastq format were firstly processed using in-house Perl scripts. Clean data were obtained by removing reads containing adapters and low-quality reads from the raw data. Transcriptome assembly was performed using Trinity software with min_kmer_cov set to 2 by default and all other parameters set default.

Assembled unigenes were annotated using the following databases: GO (http://www.geneontology.org), KEGG (http://www.genome.jp/kegg/)^26^, COG (http://www.ncbi.nlm.nih.gov/COG/), KOG (http://www.ncbi.nlm.nih.gov/KOG/), eggNOG4.5 (http://eggnogdb.embl.de/), Swiss-Prot (http://www.uniprot.org/), Pfam (http://pfam.xfam.org/) and nr database (ftp://ftp.ncbi.nih.gov/blast/db/) using BLAST tool with e-value < 1e−5.

Differentially expressed genes in the two libraries were identified using DESeq2 R package (https://bioconductor.org/packages/release/bioc/html/DESeq2.html). *p*-value corrected by Benjamini–Hochberg method^27^, “*p*-value < 0.01 and fold change ≥ 2” were used as the criteria for screening differentially expressed genes (DEGs). GO enrichment analysis was achieved by the topGO R package based on the Kolmogorov–Smirnov test. KEGG pathway enrichment was performed by KOBAS 2.0 software with FDR ≤ 0.05.

### Identification and cloning of *FT* genes from *A. konjac*

*FT* genes were retrieved from unigene annotation of the two cDNA libraries derived from *A. konjac* buds. Total RNA was extracted from *A. konjac* using HiPure HP Plant RNA Kit (Magen, China). RNA samples were reverse transcribed to cDNA using ReverTra Ace^®^ qPCR RT Master Mix (Toyobo, Japan). Full-length coding sequences of *A. konjac* FT genes were amplified with Golden Star T6 Super PCR Mix (TsingKe, China). The thermocycling conditions were as follows: denaturation of cDNA at 98 °C for 2 min, 32 cycles of 98 °C for 10 s, 60 °C for 10 s, and 72 °C for 5 s. PCR products were cloned into pClone007 Versatile Simple Vector (TsingKe, China) and sequenced at TsingKe Technologies Co, LTD. Phylogenetic analysis was performed using MEGA 7 software^28^. Conserved motifs in protein sequence were identified using MEME tool (http://meme-suite.org/) with default parameters^29^.

### Preparation of *AkFT1* and *AkFT2* overexpression lines

*AkFT1* and *AkFT2* gene were cloned into pEGAD vector (sustained in our laboratory) at the AgeI and SmaI site using ClonExpress II One Step Cloning Kit (Vazyme, China). Sequencing was performed at TsingKe Technologies Co, LTD to confirm that the genes were cloned successfully. Expression of *AkFT1* and *AkFT2* gene was modulated by *CaMV35S* promoter. *Arabidopsis thaliana ecotype* Columbia plants were transformed using *Agrobacterium tumefaciens* strain, GV3101 harboring *AkFT1* and *AkFT2* overexpression vector using the floral dip method^30^.

A total of 8 individual transgenic lines carrying *35S::AkFT1* and *35S::AkFT2* were established, respectively. Among the two transgenic materials, we selected three independent transgenic lines for analysis, and at least 40 plants were selected for observation and statistical analysis.

### Real‑time quantitative and semi-quantitative RT-PCR analysis

cDNA sequences of *A. konjac* were obtained as described above. *AkEF1-α* was used as the internal reference gene^12^ and eight DEGs genes were selected for qRT-PCR analysis. qRT-PCR was performed using ChamQ Universal SYBR qPCR Master Mix (Vazyme, China). The PCR program was: 95 °C for 30 s, then 40 cycles of 95 °C for 10 s and 60 °C for 30 s. All amplification reactions were performed in three replicates. Relative expression of each gene was determined by 2^−∆∆CT^ method^31^.

Total RNA was extracted from *A. thaliana* using TRIzol Reagent (CWBIO, China). RNA was then reverse transcribed to obtain cDNA. Further, 2 × Taq Master Mix (Dye Plus) (Vazyme, China) was used for semi-quantitative RT-PCR analysis using the following thermocycling conditions: denaturation of cDNA at 95 °C for 3 min, 28 cycles of 95 °C for 15 s, 60 °C for 15 s, and 72 °C for 30 s. *A. thaliana Actin2 *(*AtACTIN2*) gene was used as the control gene. Sequences of the gene-specific primer sets are presented in Table 1.

**Table 1:** Sequences of specific primers.

Name	Forward	Reverse
EF1-α	AAGTTCCTGAAGAATGGCGAT	GTCCCTCACGGCAAACCTACC
GA2ox	GTCAACCTCCACGCCAAGCAT	CCAGCCTCGACATTACGTCCAG
AkFT1	CGTCACCAATGGCTCCGAGTTC	TCCACCAGCACGAGTGTGTAGG
AkFT2	TGGACCCCTTCACAAGGACT	GGTCCTGAGATCGTTGCCTC
WUS	GGTAATGGCTGTGGTGGCTCTG	CGGCATTGCTGTTGGCTCCA
EJ2	CAATCGCCAACCTGCTCACTCA	TGGGCAAGTGTTTCTGGGTTCA
GAI1	ATCGGCTCAGCAGCAGCAGTA	TGATGAGGCGGAGGCAATGGT
AGL30	TCTTGGTCTGGCCGAAGGACT	GCACCGCCAAAGAAGGTAGAGA
SPL16	ACTGCTACACCAGAGCCCAACT	CCCGACAGAAGGACCCAGGAAA
AtActin	GGTGATGGTGTGTCT	ACTGAGCACAATGTTAC
AkFT1-cDNA	ATGAATAAGAGCAGTAGCAGCACCG	CTATGTGAACCTTCTTCCACCGGAAC
AkFT2-cDNA	ATGCCTCGCGAGAGGGATCCCTTGG	CTACATCCTTCTCCCGCCGGAGC
pE-AkFT1	ggggactctagcgctaccggtATGAATAAGAGC AGTAGCAGCACC	tccaagcttctcgagcccgggCTATGTGAACCTTC TTCCACCGG
pE-AkFT2	ggggactctagcgctaccggtATGCCTCGCGAG AGGGAT	tccaagcttctcgagcccgggCTACATCCTTCTCC CGCCG

### Ethics approval and consent to participate

We confirm that the collection of plant material did not involve any endangered or protected plant species and declare that the work reported here is consistent with the IUCN Policy Statement on Research Involving Species at Risk of Extinction and the current laws of China.

## Results

### Transcriptome analysis

**Figure 1:**
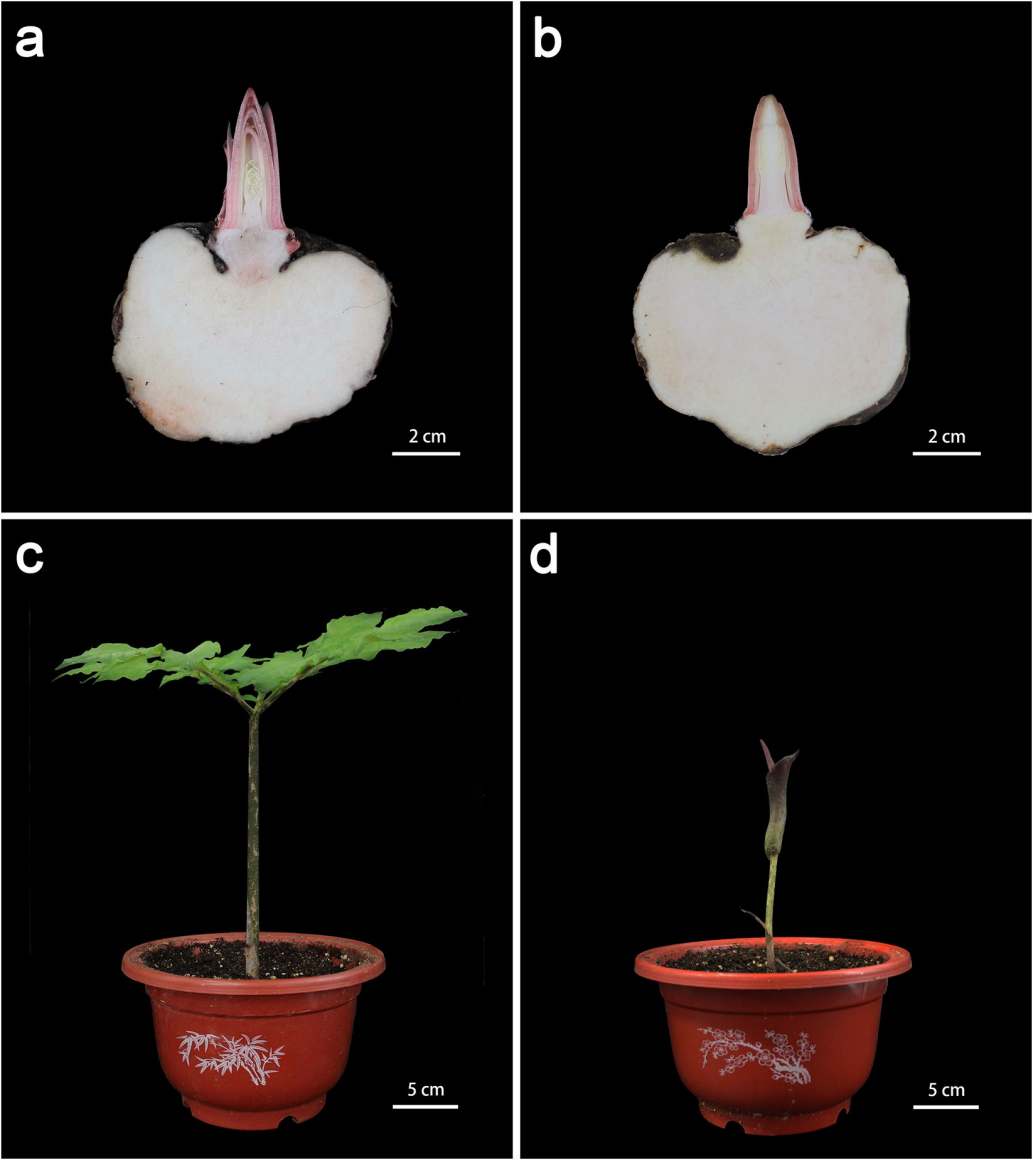
Leaf and flower of *A. konjac.* (**a**) Leaf bud. (**b**) Flower bud. (**c**) Leaf. (**d**) Flower.

*A. konjac* is a unique plant with a single petiole and a compound leaf at the top whereby the leaf and flower do not appear concurrently. Therefore, vegetative or reproductive growth of *A. konjac* is initiated through leaf buds or flower buds (Fig. 1). Two cDNA libraries were constructed from leaf primordium (LB) and flower primordium (FB) of *A. konjac* to explore gene expression during the development of leaf buds and flower buds, resulting in 48.52 and 47.95 million raw reads in the two cDNA libraries, respectively. After filtering adapters and low-quality reads from the raw data, approximately 47.79 and 47.23 million clean reads were obtained in the two cDNA libraries. Further, all clean reads were de novo assembled using Trinity software^32^ and 68,906 unigenes were obtained from the two cDNA libraries. The average size of unigenes was 920 bp, and the N50 length of unigenes was 1403 bp (Table 2). About 89.11% of unigenes had a length ranging from 300 to 2000 bp.

**Table 2:** Summary of *A. konjac* buds transcriptome data.

Statistics	Values
Total number of clean reads	95,021,826
Total number of transcripts	192,902
Mean length of transcripts	1296
N50 length of transcripts	1910
Total number of unigenes	68,906
Mean length of unigenes	920
N50 length of unigenes	1403

Unigenes were analyzed using GO, KEGG, COG, KOG, eggNOG, Swissprot, Pfam and nr databases to identify key functions of the genes. A total of 24,622 unigenes were annotated, representing only one third of the total number of unigenes. The number of unigenes annotated in each database was then determined (Table 3). Notably, 24,246 unigenes were annotated in nr database, accounting for 35.19% of the total number of unigenes, whereas only 6739 unigenes had annotation information in COG database. Functional annotation of genes expressed in leaf buds and flower buds further enriched the gene pool of *A. konjac*.

**Table 3:** Function annotation of *A. konjac* buds transcriptome.

Database	Number	Percentage
GO	12,896	18.72
KEGG	8159	11.84
COG	6739	9.78
KOG	14,047	20.39
eggNOG	22,433	32.56
Swissprot	15,692	22.77
Pfam	17,562	25.49
nr	24,246	35.19

### Differential expression analysis of genes in leaf buds and flower buds

**Figure 2:**
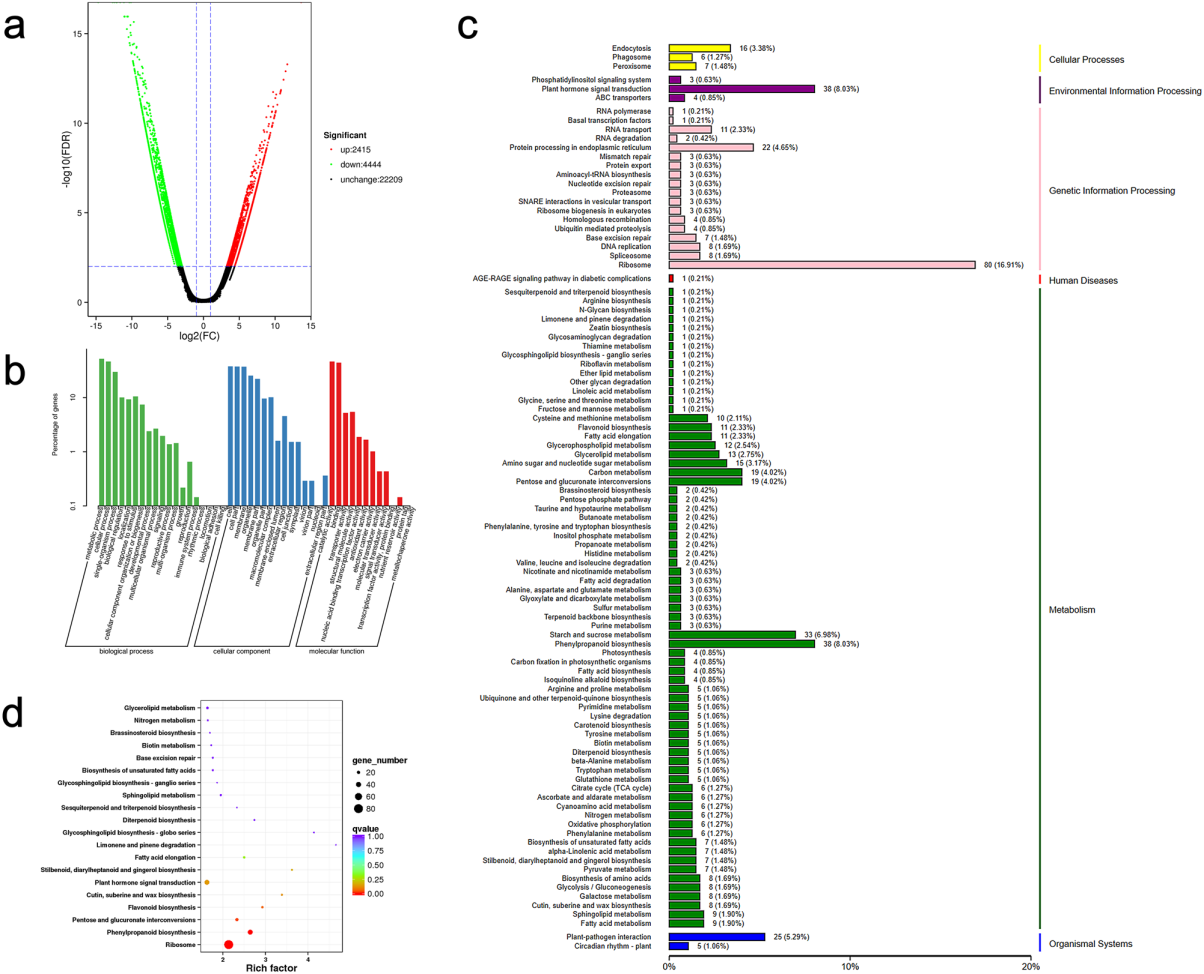
Number and functional annotation of DEGs. (**a**) Volcano plot. (**b**) GO classification. (**c**) KEGG class. (**d**) KEGG pathway enrichment map.

With LB as the control, 6859 significant DEGs were selected with 2415 DEGs were upregulated whereas 4444 DEGs were downregulated (Fig. 2a). Functional annotation was performed on the identified DEGs according to the expression levels of the genes in two libraries. A total of 2908 DEGs had annotation information (Table 4). Analysis showed that the nr database had the highest number of DEGs with annotated information with 2842 DEGs.

**Table 4:** Functional annotation of DEGs.

Database	GO	KEGG	COG	KOG	eggNOG	Swiss-Prot	Pfam	nr	All
Number	1381	862	665	1418	2556	1880	1858	2842	2908

GO functional analysis showed that 1381 DEGs were enriched in 44 classes of three major categories (biological process, cell composition and molecular function) (Fig. 2b). Some DEGs (672) were annotated as metabolic processes which was the most representative class under the “biological process” category. Some DEGs (517) were annotated as “cell part”, which was the most significantly enriched term in “cellular component” category. Under “molecular function”, DEGs were mainly involved in “binding” (611) and “catalytic activity” (639) processes. TopGO software was used to explore enrichment of DEGs^33^. The top 10 GO terms with the significant enrichment of DEGs are presented in Table 5. The top 3 GO terms included DNA integration, structural constituent of ribosome and RNA-dependent DNA biosynthetic process.

**Table 5:** Top 10 significant enrichment of GO function annotation.

Serial no.	GO.ID	Term
1	GO:0015074	DNA integration
2	GO:0003735	Structural constituent of ribosome
3	GO:0006278	RNA-dependent DNA biosynthetic process
4	GO:0003964	RNA-directed DNA polymerase activity
5	GO:0005840	Ribosome
6	GO:0005576	Extracellular region
7	GO:0022625	Cytosolic large ribosomal subunit
8	GO:0004523	RNA–DNA hybrid ribonuclease activity
9	GO:0022627	Cytosolic small ribosomal subunit
10	GO:0045330	Aspartyl esterase activity

Moreover, 862 DEGs were annotated into 98 metabolic pathways in KEGG pathway analysis (Fig. 2c). The most significantly enriched pathways were ribosome (80 DEGs), plant hormone signal transduction (38 DEGs) and phenylpropanoid biosynthesis (38 DEGs). Some DEGs (38) were implicated in plant hormone signal transduction and may be involved in flower bud differentiation and floral organ development of *A. konjac*, and studies should further explore their functions. These metabolic pathways provide a molecular foundation for studying the specific processes involved in leaf bud and flower bud development of *A. konjac*. KEGG pathway enrichment analysis showed that the top 20 pathways associated with high number of DEGs included ribosome, phenylpropanoid biosynthesis and the pentose and glucuronate interconversions (Fig. 2d). These metabolic pathways are mainly involved in synthesis of organic matter and energy transfer, indicating that there are some differences in material and energy requirements between leaf bud and flower bud development.

### DEGs related to flowering

Genes implicated in gibberellin synthesis or flowering signaling pathway were significantly differentially expressed in the two transcriptomes. *c75063.graph_c0* and *c82483.graph_c0* are homologous to *GAI* gene and their expression was downregulated in flower buds. The log_2_ (fold change) of the two *GAI* genes was − 6.47 and − 6.70, respectively. *c76528.graph_c0* which was homologous to *GA20ox* gene was highly expressed in flower buds. The log_2_ (fold change) of *GA20ox* gene was 8.26. Expression level of *c63309.graph_c0* and *c74067.graph_c0* which were homologous to *GA2ox* was lower in flower buds compared with the expression level in leaf bud. The log2 (fold change) of the two *GA2ox* genes was − 3.77 and − 6.21, respectively. 4 *SPL* homologous genes were identified from DEGs and their expression level was significantly high in flower buds relative to the expression level in leaf buds. *c73015.graph_c0* and *c100034.graph_c0* were homologous *FT* genes and had high expression level in flower buds. The log_2_ (fold change) of the two *FT* genes was 4.73 and 7.53, respectively. Nine MADS-box transcription factor genes were identified from DEGs, with 7 upregulated genes and 2 downregulated genes in flower buds. Notably, several DEGs which showed specific expression profile in flower buds were implicated in floral development.

### Other phytohormonal-related gene expression differences during flowering of *A. konjac*

Phytohormones participate in a variety of physiological and biochemical processes, and are involved in regulation of growth and development of plants. Expression patterns of phytohormone biosynthesis and signal transduction related genes in leaf buds and flower buds were analyzed to determine the regulatory effect of other phytohormones except GA on the flowering of *A. konjac*. Expression of auxin biosynthesis gene, *YUCCA4*; auxin transporter protein gene, *AUX1*; auxin responsive genes *IAA9, IAA10* and *IAA13* was upregulated in flower buds*.* In addition, expression of *cytokinin dehydrogenase* gene, *CKX5* was upregulated in flower buds compared with the expression level in leaf buds. Cytokinin response factor gene, *AHP* was downregulated in flower buds relative to leaf buds. ABA receptor gene, *PYL4* and ABA biosynthesis related genes such as *CCD8B* and *NCED1* were downregulated in flower buds compared with leaf buds. Ethylene (ETH) biosynthesis related genes including *ACS1*, *ACS3* and *ACS9* were upregulated. The gene that encodes the rate-limiting enzyme in Brassinosteroid (BR) biosynthesis, *DET2* was upregulated. Jasmonates acid (JA) biosynthesis related gene, *4CLL6*, and repressor of JA responses including *TIFY9* and *TIFY10A* were downregulated in flower buds relative to the expression level in leaf buds. Differential expression of these genes implied that these phytohormones play complex and different roles in development of leaf and flower buds. Further studies should explore the potential regulatory mechanisms.

### Verification of expression of DEGs related to flowering of *A. konjac*

**Figure 3:**
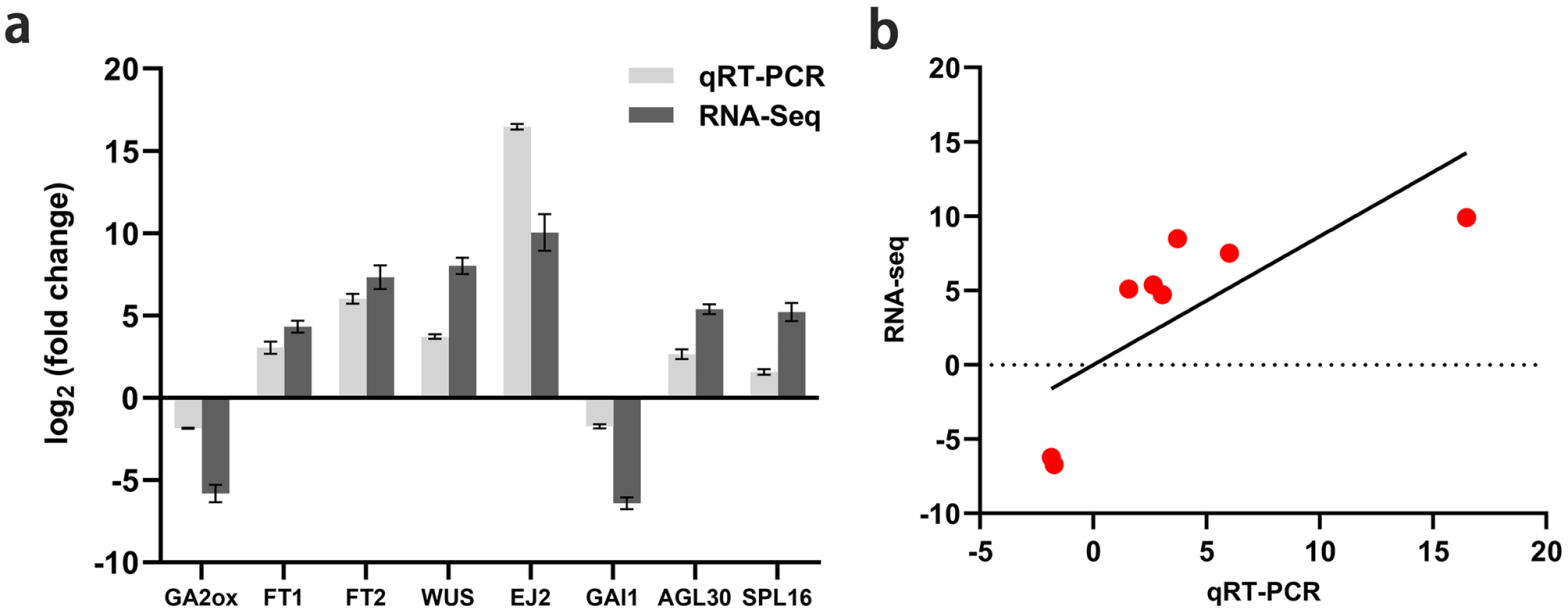
Verification of DEGs related to flowering and correlation analysis. (**a**) Verification of DEGs using qRT-PCR. The qPCR section was plotted with the expression of genes in leaf buds as a control and the relative expression of genes in flower buds, and the RNA-seq section was plotted with the leaf bud transcriptome as a control and the log2 (fold change) of genes. (**b**) Pearson correlation analysis of the expression of DEGs between qRT-PCR and RNA-Seq.

Eight DEGs implicated in flowering, of which six genes were upregulated and two genes were downregulated, were used for verification of expression levels obtained from transcriptome analysis. The results of quantitative real time-PCR were in agreement with transcriptome analysis results (Fig. 3), indicating that the transcriptome results were reliable.

### Identification and genetic analysis of *FT* genes in *A. konjac*

**Figure 4:**
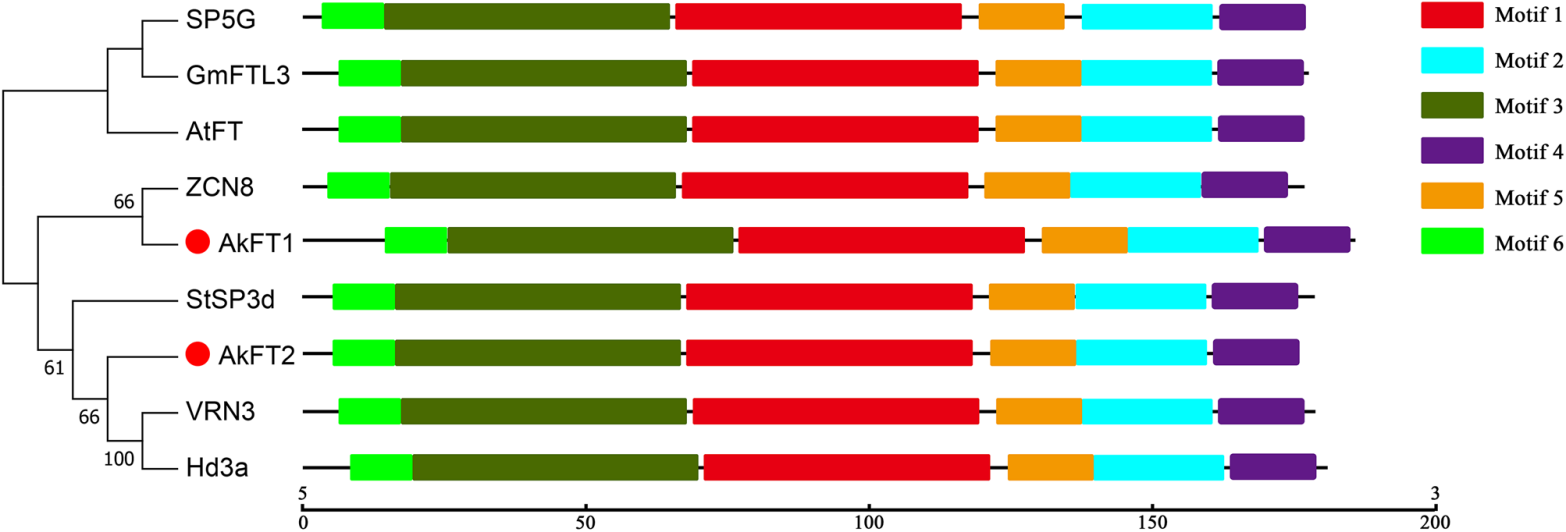
Phylogenetic analysis of FT proteins. Phylogenetic tree of FT homologous proteins using the neighbor-joining method by MEGA 7 (Bootstrap: 1000 replicates). Conservative motif analysis of FT homologous proteins using MEME. The proteins are as follows: *A. thaliana AtFT*, AAF03936.1; Rice *Hd3a*, BAO03048.1; Wheat *VRN3*, ABK32208.1; *Glycine max GmFTL3*, ACA24487.1; Maize *ZCN8*, NP_001106247.1; Tomato *SP5G*, NP_001307981.1; Potato *StSP3d*, BAV67096.1.

Two candidate genes encoding PEBP protein, *c73015.graph_c0* and *c100034.graph_c0*, were selected from the DEGs of leaf buds and flower buds of *A. konjac.* Specific PCR primers were designed to amplify the two candidate genes, respectively. *c73015.graph_c0* comprised an ORF with 555 bp, and prediction showed that it encoded for 184 amino acids. *c100034.graph_c0* comprised an ORF with 525 bp encoding 174 amino acids. The deduced protein sequences encoded by the two candidate genes were compared with other functional FT protein sequences, through phylogenetic analysis using MEGA 7 software^28^ (Fig. 4). The results showed that c73015.graph_c0 clustered with ZCN8, and c100034.graph_c0 clustered with Hd3a and VRN3. This indicates that PEBP protein of *A. konjac* was evolutionary related to PEBP proteins from monocots such as rice, wheat and maize.

Conserved motifs in these proteins were analyzed using MEME tool^29^ to further explore the function of the two FT homologous proteins (Fig. 4). The findings showed that the proteins shared motifs, indicating that FT protein structure is highly conserved in plants, and they have similar functions. These two FT homologous proteins are implicated in promoting flowering of *A. konjac*. Notably, *c73015.graph_c0* was named *AkFT1* and *c100034.graph_c0* was named *AkFT2* according to the above results.

### Functional analysis of *AkFT* genes expressed in *A. thaliana*

**Figure 5:**
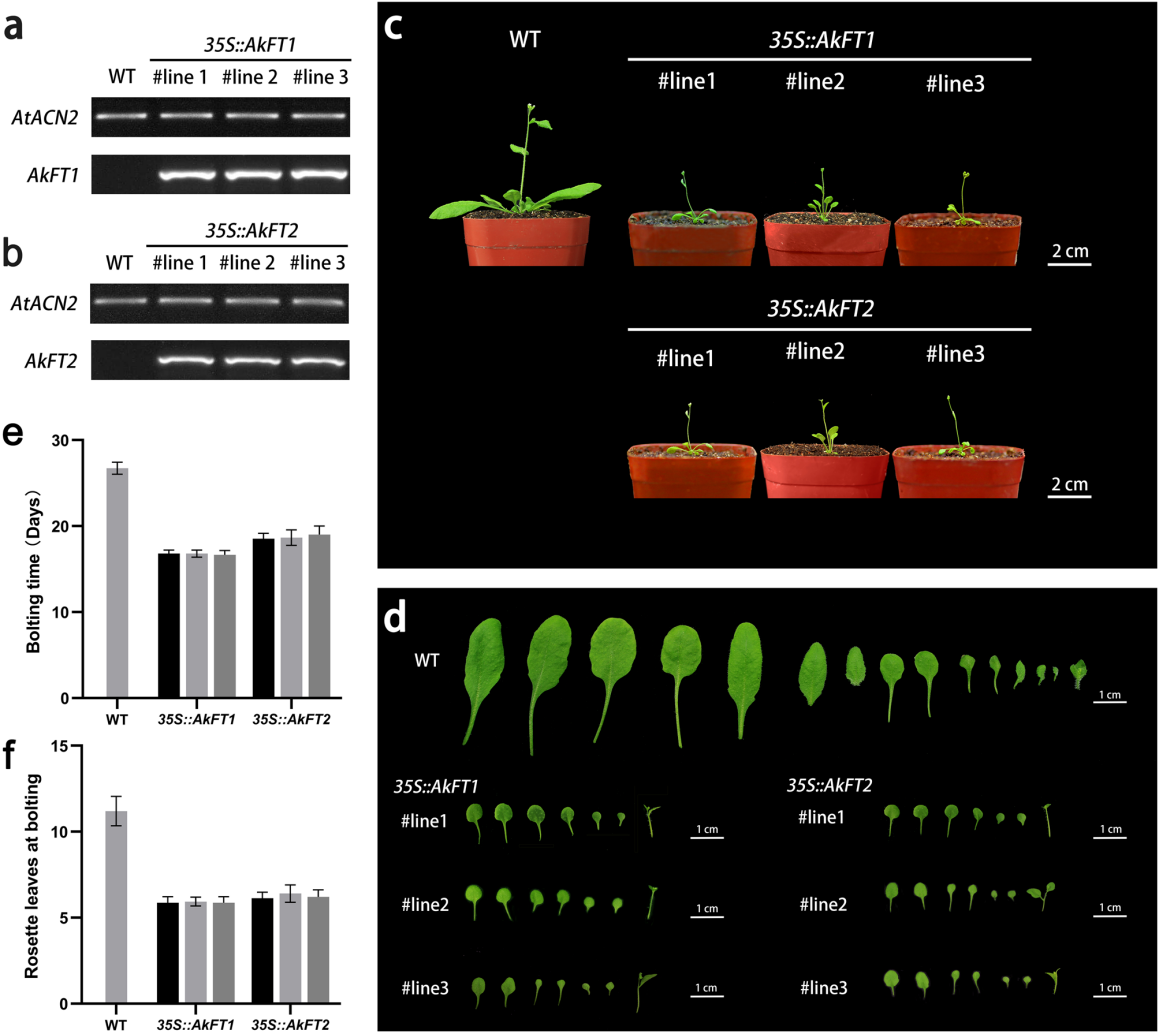
Overexpression of the *AkFT* genes in *A. thaliana.* (**a**,**b**) Semi-quantitative PT-PCR analysis of the *AkFT* genes in WT and transgenic plants. (**c**,**d**) Phenotypic observation of flowering in WT and transgenic plants. (**e**,**f**) Flowering time statistics in WT and transgenic plants. When the bolting length reached 1 cm, it was recorded as the beginning of bolting. The number of rosette leaves per plant during bolting was counted, n ≥ 40.

Phenotypic and flowering time analyses were performed on WT and transgenic plants (Fig. 5). The flowering statistics of 8 individual transgenic lines are shown in Supplementary Fig. S1 online. Semi quantitative RT-PCR results showed that *AkFT1* and *AkFT2* high expression levels in transgenic plants, respectively, but not in WT plants (Fig. 5a,b). Rosette leaves of the two types of transgenic plants was smaller and the vegetative growth period was shorter compared with those of WT plants (Fig. 5c,d). Flowering time of WT plants was 25–28 days, and 10–12 rosette leaves were observed during bolting. The number of rosette leaves ranged from 5 to 6 during the bloom period and the flowering time was about 16 days in the *35S::AkFT1* transgenic plants. Moreover, the *35S::AkFT2* transgenic plants took 18–20 days to bloom, and showed 6–7 rosette leaves at this period (Fig. 5e,f). These results indicated that *AkFT1* and *AkFT2* may play a role in promoting flowering of *A. thaliana*, respectively.

## Discussion

*A. konjac* is a unique plant whereby flowers and leaves do not occur simultaneously under natural conditions. The molecular mechanism of flower transition and development in *A. konjac* has not been fully elucidated. Studies in *A. thaliana* and other model plants indicate that at least six flowering pathways are involved in molecular control of flowering transition. Plants flower transition is controlled by growth and environmental factors through photoperiod, vernalization, autonomous, thermo sensory, gibberellin and age pathways^20,34–37^. These pathways transmit signals that regulate expression of *FT, SUPPRESSOR OF OVEREXPRESSION OF CO1 *(*SOC1*),* LEAFY* (*LFY*) and other flowering integration factors. These factors then activate the floral meristem identity genes, such as *APETALA1*(*AP1*),* FRUITFULL*(*FUL*) and other MADS box genes thus promoting floral primordia differentiation and flower organ development^38,39^. FT in *Arabidopsis* is synthesized in the leaf during the day then it is transported into meristem and functions as a co-transcription factor^19^. Therefore, FT is referred as a florigen. Regulation of the duration of light when corm is in the dormancy period can affect flowering in *A. bulbifer*^8^. However, corms mainly grow under the soil, and photoperiod may not be the main pathway that affects flowering of *A. konjac*. Exogenous gibberellin application during corm dormancy accelerates *A. muelleri* blooming^3,40^, indicating that gibberellin pathway is implicated in regulating *Amorphophallus* flowering. In addition, the corm age affects its flowering pattern. In the present study, gene expression profile of *A. konjac* leaf buds and flower buds was explored by transcriptome analysis and several DEGs were implicated in gibberellin synthesis and signaling.

We obtained 68,906 unigenes from the leaf bud and flower bud transcriptomes, of which only 24,622 unigenes (35.73%) had functional annotation information. The absence of annotation information for some genes may be due to insufficient genome-wide information, limitations of transcriptome analysis, and incomplete information in functional databases. These genes may have unique functions in konjac and deserve further research. A total of 6859 DEGs were identified by comparing the transcriptome of *A. konjac* leaf buds and flower buds and 2908 DEGs had functional annotation information. Lack of annotation information of several DEGs can be attributed to lack of reference genome and incomplete database information of *A. konjac*. This indicates that leaf buds and flower buds in *A. konjac* exhibit different and unique processes and pathways. Several key genes involved in flowering and the possible regulatory pathways in *A. konjac* were analyzed. Gibberellin homeostasis in plants is achieved by strict regulation of the activities of “activating enzymes” (GA20ox and GA3ox) and “inactivating enzymes” (GA2ox)^41,42^. *GA20ox* showed low expression levels in leaf buds, however, its showed high expression levels in flower buds. Two *GA2ox* genes were significantly downregulated in flower buds compared with the expression level in leaf buds. These results indicated that synthesis and degradation of gibberellin significantly affects flowering of *A. konjac.* DELLA protein is a negative regulator of gibberellin signaling pathway, and a member of GAI‐RGA‐and‐SCR (GRAS) family^43,44^. *A. thaliana* expresses five DELLA proteins, including GAI, RGA (REPRESSOR OF ga1–3), RGL1 (RGA-LIKE 1), RGL2 and RGL3^45,46^. Two *GAI* homologous genes were downregulated or even not expressed in flower buds, which may slow flowering of *A. konjac*. *SPL* genes regulate the flowering time of *A. thaliana* through DELLA-dependent and DELLA-independent pathways. Notably, interaction between *SPL* genes and miR156 can affect flowering through the age pathway^47,48^. Four *SPL* homologous genes showed high expression level in flower buds, implying that these genes are implicated in promoting the flowering of *A. konjac.* The expression product of *DEHYDRATION-RESPONSIVE ELEMENT-BINDING PROTEIN3* (*DREB3*) is an AP2/EREBP-type transcription factor and overexpression of *DREB3* delays flowering in tobacco^49^. *DREB3* was highly expressed in leaf buds, but showed low expression levels in flower buds, indicating that *DREB3* is a negative regulator of flowering. Most floral meristem identity genes and floral organ identity genes are members of MADS-box transcription factor family^50,51^. Nine MADS-box genes were identified from DEGs with seven upregulated genes and two downregulated genes in flower buds, implying that different MADS-box genes have different expression patterns and may play different roles in flower development. However, further studies should explore the specific mechanism.

In addition to Gibberellin, other phytohormones affect the flowering of *A. konjac* through complex regulatory mechanisms. Previous studies report that auxin plays a key role in development of inflorescence, flower meristem and flower organs^52–54^. Cytokinins modulate initiation and development of reproductive organs^55,56^. ETH is involved in regulation of the flowering time of plants^57^. In the current study, significant differences were observed in the expression profiles of several important genes related to auxin, cytokinins, ABA, ETH, JA and Br biosynthesis and signal transduction in leaf and flower buds. Notably, *YUCCA4* (Auxin biosynthesis), *CCD8B* (ABA biosynthesis), *NCED1* (ABA biosynthesis), *ACS1* (ETH biosynthesis), *ACS3* (ETH biosynthesis), *ACS9* (ETH biosynthesis), *DET2* (BR biosynthesis), *4CLL6* (JA biosynthesis) were differentially expressed in leaf and flower buds. Differential expression of genes implicated in plant hormone signal transduction affects expression of responsive genes, which may ultimately affect the flowering of *A. konjac*.

The *FT* gene encodes Phosphatidyl Ethanolamine-binding Protein (PEBP) protein which is a plant flowering integration factor^58,59^. Currently, *FT* genes from different plants have been reported^21,22,25,60–62^. Some plants have several types of *FT* genes, and their functions are different. Previous studies report that *FT* can integrate signals from various flowering pathways to promote plant flowering^63–66^. *FT-like* and *TERMINAL FLOWER 1*(*TFL1*)-*like* genes affect several physiological processes in plants, such as seed development and germination in *A. thaliana*^23,67^, corm formation of potato^68^ and bulb development of onion^24^. In this study, two FT homologous genes were isolated from flower buds of *A. konjac* and were named *AkFT1* and *AkFT2*. Sequence alignment showed that the two FT proteins are high homologous to several plant FT proteins and belonged to FT-like protein. Phylogenetic analysis showed that these genes were highly related to FT protein expressed in monocotyledons, such as rice, wheat and maize. The two FT homologous proteins shared conserved motifs with several FT proteins, indicating that they may have similar biological functions. *AkFT1* and *AkFT2* were significantly upregulated in flower buds relative to the expression level in leaf buds. This finding was further confirmed by qRT-PCR analysis, indicating that they may be positive regulators of flowering in *A. konjac*. Significantly, FT protein is transported via the phloem for a long distance, and finally lead to the formation of flowers in the shoot apex^17,69^. This unique feature of *A. konjac* leaf and flower not developing simultaneously, and the detection of *AkFT1* and *AkFT2* mRNAs in flower buds, suggests that the expression pattern of FT in Konjac differs from that of other plants. Overexpression of *AkFT1* and *AkFT2* reduced the vegetative growth period of *A. thaliana* and accelerated flowering compared with the wild type plants. *AkFT1* and *AkFT2* genes may play a critical role similar to the function of florigen during flowering transition of *A. konjac,* however, the precise molecular mechanism should be explored further.

In summary, comprehensive gene expression information of leaf buds and flower buds of *A. konjac* was obtained through transcriptome analysis. These results showed that some genes are differentially expressed during the development of leaf buds and flower buds. Two *FT* homologous genes (*AkFT1* and *AkFT2*) were identified, which exhibited high expression level in flower buds relative to the expression level in leaf buds. Overexpression of *AkFT1* and *AkFT2* significantly decreased flowering time of transgenic *A. thaliana* relative to the wild type plants.

## Supplementary Information

Supplementary Figure S1.

Supplementary Figures.
