# Supplementary figures and images for: Comparative analysis of buds transcriptome and identification of two florigen gene AkFTs in Amorphophallus konjac
Source: Sci Rep. 2022 Apr 26;12:6782. doi: 10.1038/s41598-022-10817-5 (PMC9043200; doi:10.1038/s41598-022-10817-5)

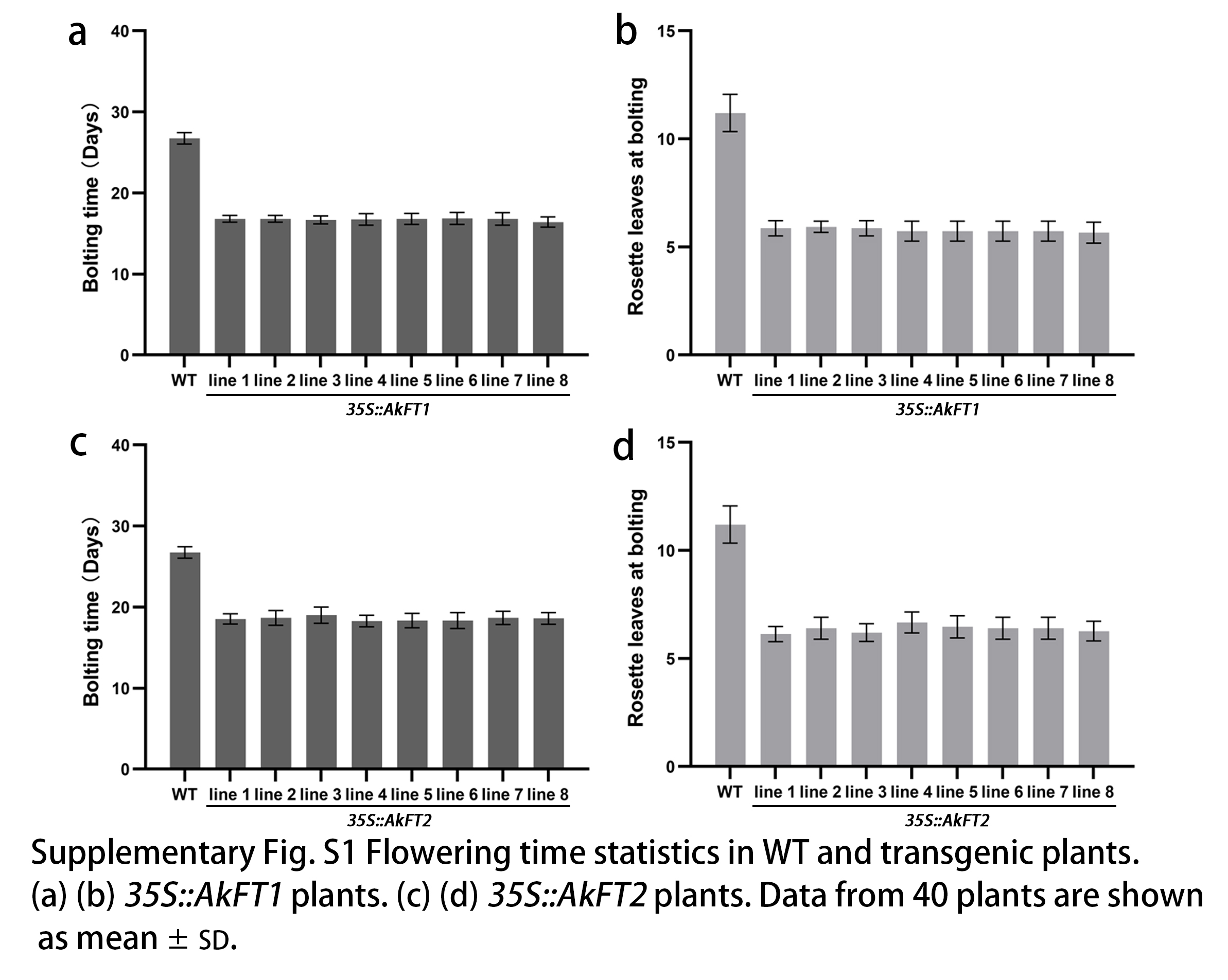

Supplement: Supplementary file 1 — Supplementary Figure S1. [file 41598_2022_10817_MOESM1_ESM.jpg]

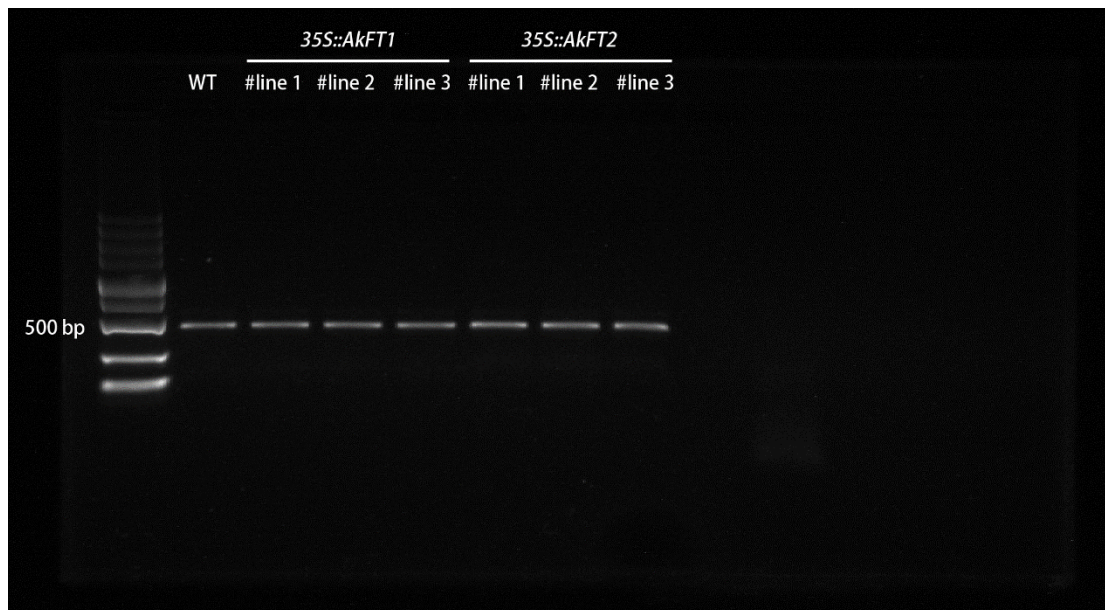

Figure. S1 Gel electrophoresis of *AtACN2*.

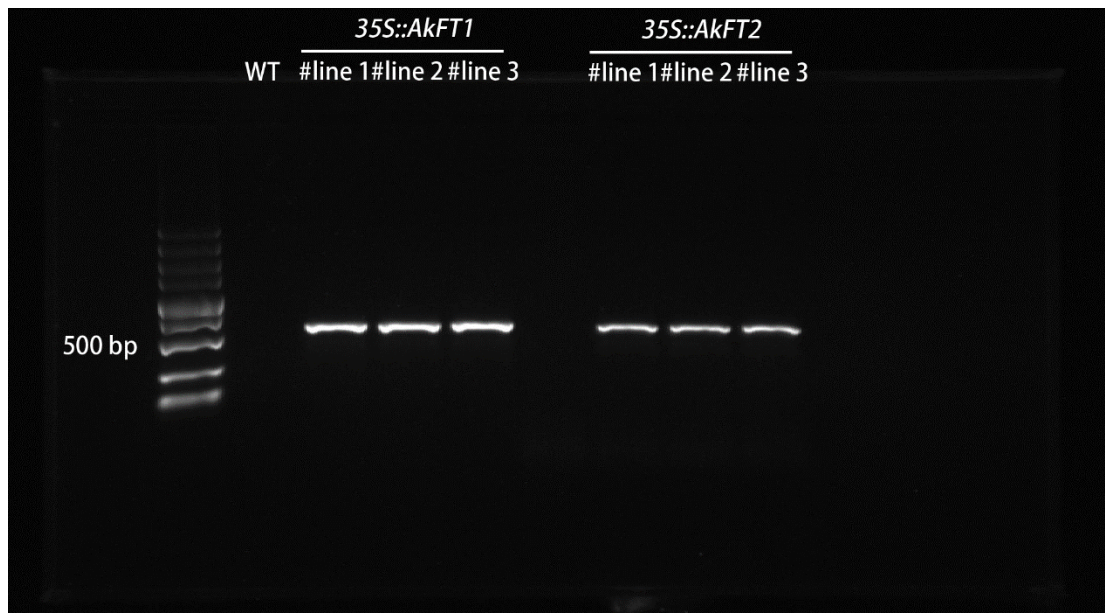

Figure. S2 Gel electrophoresis of *AkFT1* and *AkFT2*.

Supplement: Supplementary file 2 — Supplementary Figures. [file 41598_2022_10817_MOESM2_ESM.pdf]
